# Distribution Characteristics and Environmental Control Factors of Lipophilic Marine Algal Toxins in Changjiang Estuary and the Adjacent East China Sea

**DOI:** 10.3390/toxins11100596

**Published:** 2019-10-12

**Authors:** Xiuping He, Junhui Chen, Danni Wu, Ping Sun, Xin Ma, Jiuming Wang, Lijun Liu, Kan Chen, Baodong Wang

**Affiliations:** 1 Key Laboratory for Marine Bioactive Substances and Modern Analytical Technology, The First Institute of Oceanography, Ministry of Natural Resources, Qingdao 266061, China; hexiuping@fio.org.cn (X.H.); wudanni@fio.org.cn (D.W.); sunping@fio.org.cn (P.S.); jmwang@fio.org.cn (J.W.); liulj01@fio.org.cn (L.L.); chenkan1225@foxmail.com (K.C.); wangbd@fio.org.cn (B.W.); 2 Laboratory for Marine Ecology and Environmental Science, Pilot National Laboratory for Marine Science and Technology (Qingdao), Qingdao 266071, China; 3 Qinhuangdao Marine Environmental Monitoring Central Station, Qinhuangdao 066000, China; maxin@ncs.mnr.gov.cn; 4 Marine College, Shandong University, Weihai 264200, China

**Keywords:** marine algae toxins, large river estuary, Changjiang estuary, East China Sea, seawater, phytoplankton

## Abstract

Marine algal toxins, highly toxic secondary metabolites, have significant influences on coastal ecosystem health and mariculture safety. The occurrence and environmental control factors of lipophilic marine algal toxins (LMATs) in the surface seawater of the Changjiang estuary (CJE) and the adjacent East China Sea (ECS) were investigated. Pectenotoxin-2 (PTX2), okadaic acid (OA), dinophysistoxin-1(DTX1), and gymnodimine (GYM) were detected in the CJE surface seawater in summer, with concentration ranges of not detected (ND)–105.54 ng/L, ND–13.24 ng/L, ND–5.48 ng/L, and ND–12.95 ng/L, respectively. DTX1 (ND–316.15 ng/L), OA (ND–16.13 ng/L), and PTX2 (ND–4.97 ng/L) were detected in the ECS during spring. LMATs formed a unique low-concentration band in the Changjiang diluted water (CJDW) coverage area in the typical large river estuary. PTX2, OA, and DTX1 in seawater were mainly derived from *Dinophysis caudate* and *Dinophysis rotundata*, while GYM was suspected to be from *Karenia selliformis*. Correlation analyses showed that LMAT levels in seawater were positively correlated with dissolved oxygen and salinity, but negatively correlated with temperature and nutrients, indicating that the hydrological condition and nutritional status of seawater and climatic factors exert significant effects on the distribution of LMATs.

## 1. Introduction

Marine phycotoxins are secondary metabolites produced by some toxigenic microalgae. Generally, the concentration of marine toxins in seawater is very low. However, the density of toxigenic algae (for example, harmful algae bloom) can increase under the influence of specific climate and environmental factors [1,2]. Increases in toxigenic algae could lead to a sharp increase in algal toxins. Ultimately, the toxic metabolites released into seawater by marine toxigenic algae present an important component in the marine system. Marine algal toxins are highly toxic organic matter that pose threats not only to marine ecosystems and marine life, but also to human health [3,4,5]. To date, hundreds of marine algal toxins have been found, and these toxins have been divided into eight categories according to their chemical structure [6,7]. Paralytic shellfish poison toxins (PSP) and domoic acid (DA) are classified as hydrophilic shellfish toxins, while other toxins with lower polarity are classified as lipophilic marine algal toxins (LMATs), mainly including okadaic acid (OA), pecenotoxins (PTXs), yessotoxin (YTXs), azaspiracid (AZAs), cyclic imines, and brevetoxin [8,9]. LMATs, which have diverse chemical structures and good stability, account for 90% of all marine algal toxins. Since LMATs can be easily absorbed and accumulated by shellfish, they can seriously affect the marine fishery and aquaculture industries [8,10,11,12].

Developments in modern analytical techniques, especially solid-phase extraction and liquid chromatography-mass spectrometry (LC-MS), have enabled the intensive study of “highly bioactive” marine algal toxins at the molecule level. Marine algal toxins, such as OA, DTXs, PTXs, YTXs, AZAs, spirolide toxins (SPXs), and gymnodimine (GYM), have been detected in the seawater of many countries, such as New Zealand [13], Australia [14], Norway [15,16], Ireland [17,18], Spain [19], France [20], China [8,10,21,22,23], and other countries [24], which indicates that LMATs exist worldwide. In Spain, OA is widely distributed in the coastal seawater at concentrations ranging from 2.10 ng/L to 1780 ng/L [19]. Chen et al. [8] found that OA, DTX1, and PTX2 are widely distributed in the surface seawater of Bohai and Huanghai in China at concentrations ranging from the limit of detection (LOD) to 55.85 ng/L, from LOD to 143.14 ng/L, and from LOD to 14.14 ng/L, respectively, thus indicating that LMATs have obvious spatial and temporal distribution characteristics [21]. As global warming, ocean acidification, and eutrophication intensify, the amounts of toxigenic dinoflagellates are expected to increase [25,26,27]. Levels of the main LMAT producers *Dinophysis* and *Prorocentrum* [28] may also increase, leading to the trend of increasing marine algal toxins [29,30]. Therefore, investigation of the composition, concentration, distribution, and sources of LMATs in typical estuaries, bays, and shellfish aquaculture areas is important for offshore marine ecological environment protection, healthy shellfish aquaculture development, and marine product safety.

Influenced by upstream runoff and eutrophication, the Changjiang estuary (CJE) and adjacent areas are among the most frequently reported to have harmful algal blooms in China. Since the 21st century, harmful algae blooms in this area have increased sharply in spring and summer [31]. As algal species that can produce harmful and toxic matter continue to increase, toxigenic algae, including *Dinophysis*, *Prorocentrum*, and *Alexandrium*, have increasingly been detected in this area [32,33]. The formation, succession, and mechanisms of harmful algal blooms are complex. The physiological and ecological characteristics of different groups of phytoplankton and environmental factors, including nutrition, temperature, illumination, and salinity, may influence harmful algal blooms [34]. The growth of toxigenic dinoflagellates, and the accumulation and release processes of toxins are also affected by various climatic and environmental factors [35]. To date, no systematic reports on the occurrence, spatiotemporal distribution, origin, and environmental control factors of LMATs in seawater of the CJE and its adjacent areas in China have been reported. The objectives of this paper are to investigate the composition, concentration, and distribution characteristics of LMATs in seawater in the CJE and its adjacent areas; probe the relationship between the concentration and distribution characteristics of LMATs in seawater and environmental factors, including temperature (T), salinity (S), pH, dissolved oxygen (DO), total suspended substances (TSS), chlorophyll a (Chla), and nutrients; and explore the source of LMATs in the marine system of the CJE and its adjacent areas.

## 2. Results and Discussion

### 2.1. Study Area and Sample Collection

The study area was located at the Changjiang estuary (CJE) of China and East China Sea (ECS) coastal area, with latitude between 25° N and 33° N, and longitude between 120° E and 125° E. The research area has complex environmental conditions and is significantly influenced by the Changjiang diluted water (CJDW), Minzhe coastal water (MZCW), and Taiwan warm current (TWWC) systems. In summer, low salinity and large amounts of sediment and nutrients in CJDW can affect the growth and reproduction of phytoplankton. The offshore branch of the TWWC extends toward the northwest near the CJE and may affect the structure of temperature (T) and salinity (S), as well as water stratification, the upwelling water, and nutrient composition, thereby influencing the biodiversity and distribution of phytoplankton in the area; even the incidence of harmful algal blooms may be affected by the CJDW and TWWC [34,36,37,38,39]. According to statistics, up until 2017, over 700 red tide outbreaks occurred in this area [40]. Therefore, the study of LMATs in the CJE and its adjacent area is of high practical significance.

**Figure 1:**
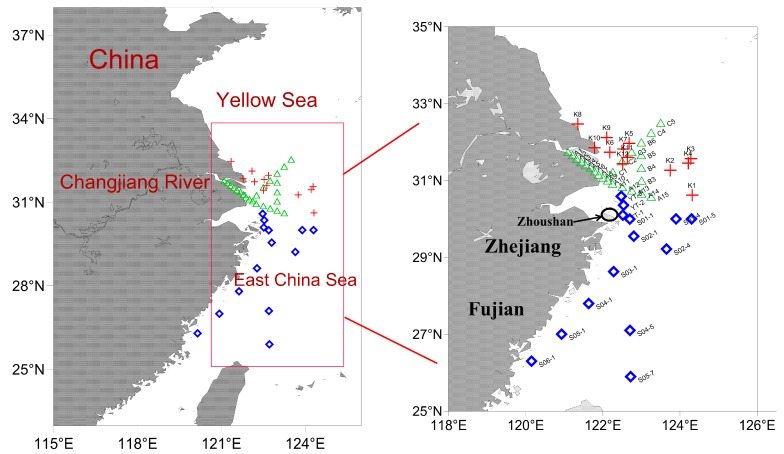
Illustration of sampling sites for seawater and phytoplankton in the Changjiang estuary and the adjacent East China Sea. Triangles represent stations in August 2017, squares represent stations in May 2018, and crosses represent stations in July 2018.

Samples were collected from the CJE and its adjacent ECS during three cruises on August 21–30, 2017, May12–21, 2018, and July13–19, 2018 (Figure 1). A total of 50 surface seawater samples and 19 phytoplankton samples were collected. Surface seawater samples were filtered using 0.7 μm GF/F glass filters after collection and stored at −20 °C until analysis. Phytoplankton samples were concentrated using vertical net hauls (20 μm mesh size, 0.25 m diameter, and 1.4 m length) from 10 m to the surface, or from the bottom to the surface if the depth was less than 10 m. Phytoplankton samples collected during August 21–30, 2017, were preserved with Lugol’s iodine (final concentration of 1%) for phytoplankton composition identification and morphological characteristics; phytoplankton cells collected during August 21–30, 2017, and May 12–21, 2018, were filtered onto a 1.6 μm glass microfiber filter (Whatman) under low vacuum and stored at −20 °C for toxin composition analysis.

### 2.2. Identification of LMATs in Seawater and Phytoplankton

**Figure 2:**
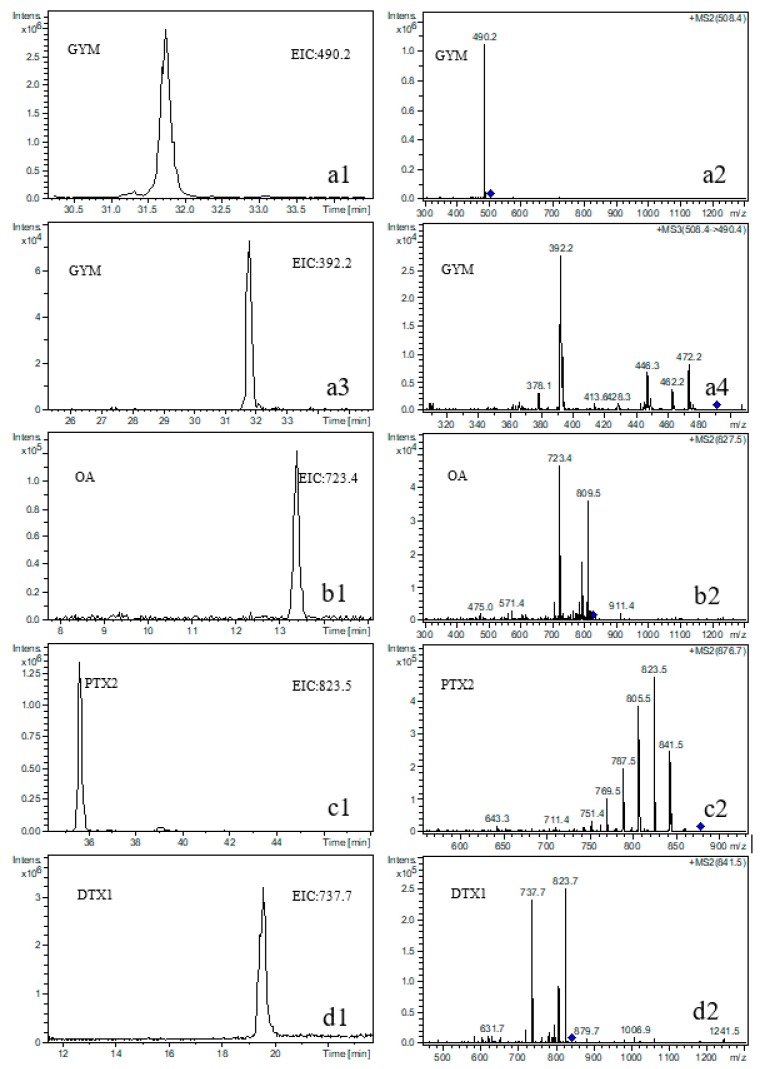
Extracted ion chromatograms (EICs) and MS/MS spectra of GYM, OA, PTX2, and DTX1 detected in surface seawater samples collected from station A15 in August 2017. (**a1**): EIC of GYM; (**a2**): MS^2^ spectrum of GYM; (**a3**): EIC of the daughter ion of GYM; (**a4**): MS^3^ spectrum of GYM; (**b1**): EIC of OA; (**b2**): MS^2^ spectrum of OA; (**c1**): EIC of PTX2; (**c2**): MS^2^ spectrum of PTX2; (**d1**): EIC of DTX1; (**d2**): MS^2^ spectrum of DTX1. Note: GYM = gymnodimine; OA = okadaic acid; PTX2 = pectenotoxin-2; DTX1 = dinophysistoxin-1.

The eight typical LMATs in seawater and phytoplankton samples were qualitatively identified and quantitatively determined using high performance liquid chromatography-tandem mass spectrometry (HPLC-MS/MS). Extracted ion chromatograms (EICs) and MS/MS spectra of these LMATs standards are shown in Figure S1 (Supplementary Materials). LMATs detected from the surface seawater of station A15 are shown in Figure 2. The process of qualitative identification of LMATs in seawater and phytoplankton is described here by taking GYM as an example. The retention time of GYM in the HPLC separation process is 31.8 min (Figure 2(a1)) and the daughter ion [M-H_2_O+H]^+^ with *m*/*z* 490 (Figure 2(a2)) was generated by GYM. To confirm the existence of GYM and obtain more qualitative information, the third-order mass spectrometry was performed. The granddaughter ions of GYM with *m*/*z* 392, 472, 462, 446, and 378 were obtained (Figure 2(a3,a4)), and found to be in accordance with our previous report [41]. Compared with the EIC (Figure S1(e1,e3) in the Supplementary Materials) and MRM results of GYM standards, including daughter (Figure S1(e2) in the Supplementary Materials) and granddaughter ions (Figure S1(e4) in the Supplementary Materials), the retention time and fragment ion peaks of GYM in seawater samples showed a small difference that was within the European Union’s regulations on the qualitative identification of compounds with low-resolution mass spectrometry [42]. Therefore, the presence of GYM in the seawater samples was definitely confirmed. OA (Figure 2(b1,b2)), PTX2 (Figure 2(c1,c2)), and DTX1 (Figure 2(d1,d2)) were also detected in the seawater and phytoplankton samples via a process similar to that applied for GYM.

### 2.3. Composition, Concentration, and Distribution Characteristics of LMATs in Seawater

**Figure 3:**
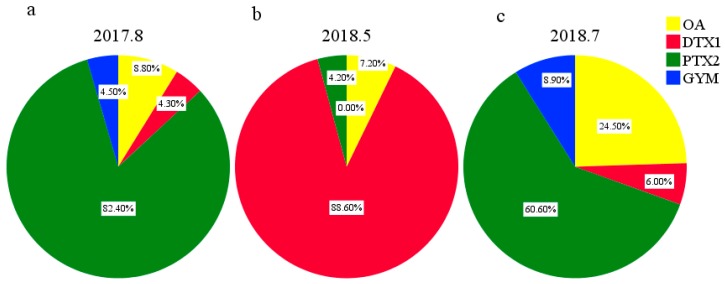
Composition of lipophilic marine algal toxins (LMATs, mass fraction) in surface seawater samples collected from the Changjiang estuary and the adjacent East China Sea during three cruises. (**a**): 2017.8; (**b**): 2018.5; (**c**): 2018.7.

Eight types of LMATs were analyzed, while only four kinds of LMATs were detected in the surface seawater collected in the CJE, and three kinds of LMATs were detected in the surface seawater collected in the ECS. The composition and concentration of LMATs in the surface seawater are shown in Table S1 (Supplementary Materials). PTX2, OA, GYM, and DTX1 were detected from the surface seawater collected in August 2017 (Figure 3a) and July 2018 (Figure 3c). PTX2, OA, and DTX1 were detected from the surface seawater collected in May 2018 (Figure 3b). In August 2017, PTX2 accounts for 82.40% of all detected LMATs (Figure 3a). Since PTX2 is the only LMAT detected in the surface seawater of stations A2 and A3, the proportion of PTX2 is 100% in stations A2 and A3 (Figure S2 in the Supplementary Material). In July 2018, the average proportion of PTX2 is 60.60% (Figure 3c) and the highest proportion of PTX2 is 70.39% (Figure S2 in the Supplementary Material). LMATs in the surface seawater of CJE were dominated by PTX2 in summer, followed by OA, GYM, and DTX1. LMATs in the surface seawater of the ECS in spring were dominated by DTX1 (average proportion: 88.60%; highest proportion: 94.98%), followed by OA (average proportion: 7.20%) and PTX2 (average proportion: 4.20%) (Figure 3b). Li et al. [43] found that OA, DTX1, and PTXs were the main toxins in mussels collected from ECS in May 2011. GYM can also be detected in mussels but exhibits the highest levels in winter. In combination with our result, we can conclude that the average proportions of different LMATs obviously varied. This paper clarifies the composition of LMATs in the surface seawater of the CJE and the adjacent ECS for the first time. Compared with the composition of LMATs in the seawater of Yellow Sea and the Bohai Sea of China [8,10], the chemical diversity of LMATs in the CJE and the adjacent ECS is more abundant.

**Figure 4:**
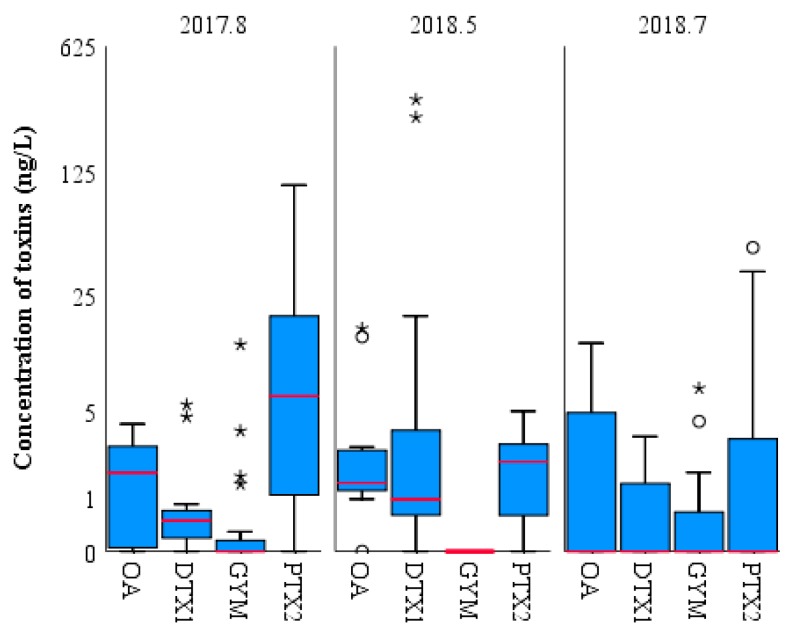
Comparison of LMAT concentrations (ng/L) in surface seawater samples collected from the Changjiang Estuary and the adjacent East China Sea during three cruises. The bold line represents the median, the box height represents the interquartile range (IQR, 25%–75%), the circles and small stars are outliers, and the black vertical line represents the range of 1.5 IQR within the maximum and minimum interquartile values.

The concentration of LMATs in the surface seawater of each station collected during three cruises is shown in Figure 4. In August 2017, the average concentration of PTX2 was 15.52 ng/L, which was the highest level among all detected LMATs. PTX2, with concentrations ranging from to 105.54 ng/L, the median value is 6.33 ng/L. The number of stations with concentrations lower than the average value was more than stations with concentrations higher than the average value. The average concentration of OA was 1.66 ng/L, and concentrations of this toxin ranged from not detected (ND) to 4.07 ng/L. No outlier was observed, as indicated in Figure 4, and the median (1.72 ng/L) was consistent with the average. This result indicates that the concentration of OA does not markedly fluctuate. The highest concentration of OA in the study area is lower than that in the coastal area of Spain (1780 ng/L) [19]. Two stations with abnormally high DTX1 concentrations were found, as indicated in Figure 4 marked with stars. Although the average concentration of DTX1 was only 0.80 ng/L, concentrations of this toxin ranged from ND to 5.48 ng/L, and its median (0.48 ng/L) was slightly lower than its average. Except the two high-value stations marked with stars, concentrations of DTX1 showed no marked fluctuation. GYM was detected in only nine of the 25 stations and exhibited high values in four stations (Figure 4, marked with stars), leading to a large difference between its median and average. Although the average concentration of GYM was 0.85 ng/L, concentrations of this toxin ranged from ND to 12.95 ng/L. The toxin showed a concentration variation tendency similar to that of DTX1. In May 2018, the average concentration of DTX1 peaked at 42.74 ng/L and concentrations of this toxin ranged from ND to 316.15 ng/L. Due to the low concentration of DTX1 at most stations and the abnormally high value at only two stations, the median (0.94 ng/L) was far lower than the average. The average concentration of OA was 3.46 ng/L, and concentrations of this toxin ranged from ND to 16.13 ng/L. The variation of OA concentration was similar to that of DTX1. The average concentration of PTX2 (2.01 ng/L) was consistent with the median (2.14 ng/L). Since no outlier was observed, no marked fluctuation in PTX2 concentration was found at each station. In July 2018, LMATs were detected in less than 50% of the seawater samples, meaning the median values were all below the middle of the interquartile range (IQR). PTX2 exhibited a maximum average concentration of 7.39 ng/L, and concentrations of this toxin ranged from ND to 47.05 ng/L. OA exhibited the second highest concentration at 2.99 ng/L, and concentrations of this toxin ranged from ND to 13.24 ng/L. The average concentrations of GYM and DTX1 were 1.08 and 0.73 ng/L, respectively, and the concentrations of these toxins ranged from ND to 7.00 ng/L and from ND to 3.33 ng/L, respectively. The average concentration of LMATs in surface seawater of the CJE is slightly higher than that in the coastal bays of the Yellow Sea and the Bohai Sea of China [8,10]. In summary, the concentration of LMATs in surface seawater of the CJE and the adjacent ECS is in the range of dozens of nanograms per liter, which is slightly higher than that in the Yellow Sea and Bohai Sea of China, and moderately lower than in some coastal areas of Europe.

**Figure 5:**
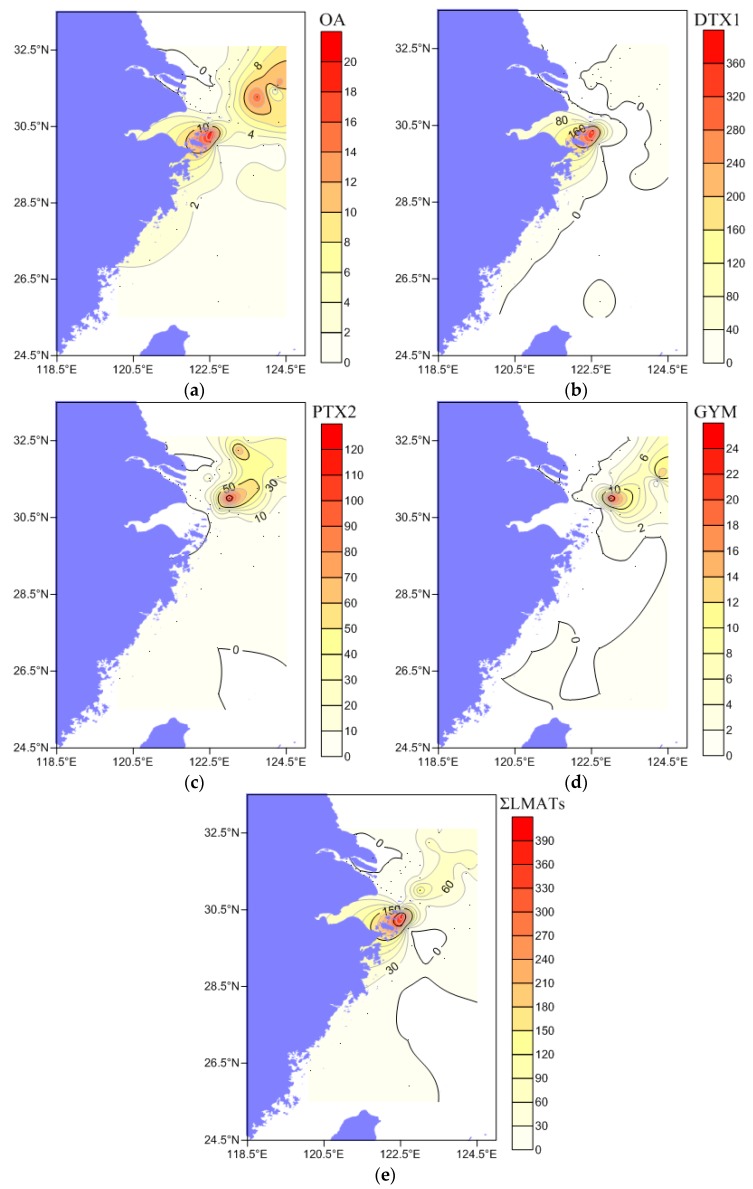
Spatial distribution characteristics of LMATs in the surface seawater of the Changjiang estuary and its adjacent East China Sea: (**a**) the distribution of OA in seawater; (**b**) the distribution of DTX1 in seawater; (**c**) the distribution of PTX2 in seawater; (**d**) the distribution of GYM in seawater; (**e**) the distribution of ∑LMATs in seawater (Unit: ng/L).

To clarify the spatial distribution characteristics of LMATs in surface seawater of the CJE and its adjacent ECS, the concentration distribution of four LMATs in the surface seawater samples obtained from three cruises is summarized in Figure 5. The concentration of OA (Figure 5a) was the highest in the YT section (from YT-1 station to YT-3 station) near the Zhoushan fishing grounds, as well as B3 and K2 stations outside the CJE. The concentration of DTX1 (Figure 5b) was the highest only in the YT section. The concentration of PTX2 (Figure 5c) was the highest at stations B3, C4 and K3, similar to GYM (Figure 5d). PTX2 was partially distributed in the coastal area of Fujian and Zhejiang (section S04), but GYM was not detected in the ECS area. The concentrations of the four LMATs in the area covered by the CJDW were relatively low. The spatial distribution of the total concentrations of LMATs (ΣLMATs) is shown in Figure 5e.

The concentration of ΣLMATs was the lowest in the area near CJDW and its northward branch. The highest concentration was found in the YT section near the Zhoushan fishery, and the second highest area was found at stations B3 and C4. This result indicates that the concentration of ΣLMATs in the surface seawater of the CJE is low in near-shore areas and high in offshore areas, consistent with the direction of the CJDW, and then decreases with increasing distance from offshore. The spatial distribution of LMATs in the surface seawater of the CJE is very different from that in the coastal areas of the Yellow Sea and Bohai Sea in China, which presents a decreasing trend with increasing distance [8,10]. This observation shows that the expansion of a large amount of CJDW into the sea affects the production, transport, and environmental fate of LMATs in the surface seawater of the CJE, eventually forming a unique low concentration zone of LMATs in the CJDW coverage area of the large river estuary. This report is the first to illuminate the spatial distribution characteristics of LMATs in the surface seawater of a large river estuary.

### 2.4. Composition and Concentration of LMATs in Phytoplankton

**Figure 6:**
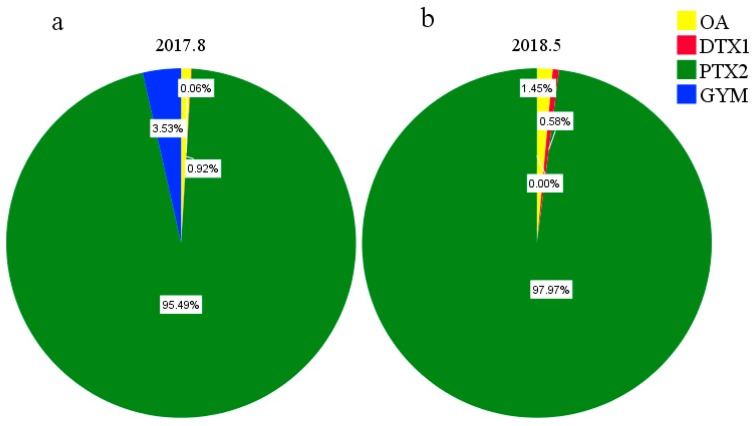
Mass fraction of LMATs in phytoplankton samples collected in August 2017 (**a**) and May 2018 (**b**).

The composition of LMATs in the phytoplankton samples collected in August 2017 and May 2018 is shown in Figure 6. All eight types of LMATs were analyzed; only four kinds of LMATs (namely PTX2, GYM, OA, and DTX1) were detected in phytoplankton samples collected in August 2017, and only three kinds of LMATs (namely PTX2, OA, and DTX1) were detected in phytoplankton samples collected in May 2018. The average proportions of PTX2, GYM, OA, and DTX1 collected in August 2017 were 95.49%, 3.53%, 0.92%, and 0.06%, respectively, while PTX2, OA, and DTX1 collected in May 2018 were detected at average proportions of 97.97%, 1.45%, and 0.58%, respectively. This showed that the composition of LMATs in phytoplankton is consistent with that in seawater. The composition of LMATs in phytoplankton from the CJE in summer was more abundant than that from the ECS in spring. The variation trends of the average proportions of various LMATs in phytoplankton samples collected from two different cruises were identical. Although the proportions of various LMATs in seawater and phytoplankton at each station slightly differed, the overall ratios were similar, as shown in Figure S3 (Supplementary Materials). In summary, although the fluctuating trend of different LMATs collected from the two cruises is similar, more LMATs are present in phytoplankton from the coastal water of the CJE in summer than from the ECS in spring. In addition, the composition of LMATs in phytoplankton is consistent with that in seawater.

**Figure 7:**
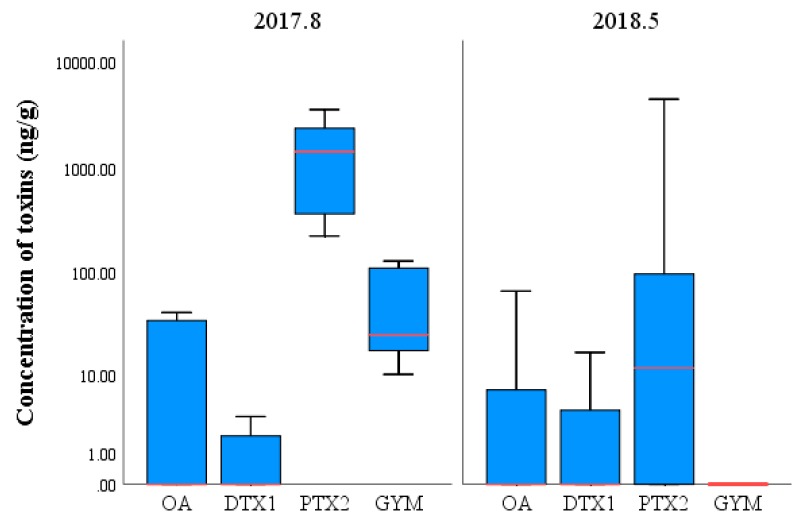
Comparison of LMAT concentrations (ng/g) in phytoplankton samples collected in August 2017 and May 2018. The bold line represents the median, the box height represents the interquartile range (IQR, 25%–75%), the circles and small stars are outliers, and the black vertical line represents the range of 1.5 IQR within the maximum and minimum interquartile values.

The concentrations of LMATs in phytoplankton samples collected from different stations in August 2017 and May 2018 are shown in Figure 7. In the phytoplankton samples collected in August 2017, the average concentration of PTX2 peaked at 1589.48 ng/g, and concentrations of this toxin ranged from 225.06 ng/g to 3565.50 ng/g. The median (1421.97 ng/g) was consistent with the average, and the variation of data at each station was not significant. The concentration of GYM reached 58.71 ng/g, and concentrations of this toxin ranged from 10.01 ng/g to 129.84 ng/g. The median (25.17 ng/g) was lower than the average. The average concentrations of OA and DTX1 were 15.24 ng/g and 1.05 ng/g, with ranges of ND–41.52 ng/g and ND–3.39 ng/g, respectively. The concentrations of OA and DTX1 in each station showed large fluctuations. In the phytoplankton samples collected in May 2018, the average concentration of PTX2 peaked at 523.84 ng/g, and concentrations of this toxin ranged from ND to 4483.15 ng/g; the median (11.79 ng/g) was much lower than the average. The concentration of PTX2 in section S01 was much higher than that in other stations. The average concentration of OA was 7.73 ng/g, and concentrations of this toxin ranged from ND to 67.05 ng/g. The concentration of OA was the highest at station S04-1. The average concentration of DTX1 was 3.11 ng/g, and concentrations of this toxin ranged from ND to 16.78 ng/g. The concentration of DTX1 was highest at station S04-1. Although the LMATs in seawater originated from phytoplankton, the proportions of different LMAT concentrations in seawater and phytoplankton differed because of differences in the intracellular and extracellular proportions of LMATs and their stabilities in seawater. Studies have shown that OA and GYM are relatively stable in seawater and cannot be degraded rapidly [44,45]. Unfortunately, no report on the stability of other LMATs in seawater is yet available. Although the average concentrations of different LMATs in phytoplankton samples collected from the two cruises exhibited the same fluctuation trend, LMAT concentrations are higher in phytoplankton collected from the CJE in summer than in those obtained from the ECS in spring. The proportions of various LMATs in phytoplankton in different sampling stations exhibited some differences compared with those in seawater.

### 2.5. Phytoplankton Community Characteristics and LMAT Origins

Species identification, cell counting, and composition analysis of phytoplankton samples collected in August 2017 were conducted using an Olympus inverted optical microscope with a 0.1 mL phytoplankton counting frame. The results are shown in Table S2 (Supplementary Materials). Diatoms were found, including 31 species from 20 genera, accounting for 53% of the total. The dinoflagellates were found, including 24 species from 15 genera, accounting for 41% of the total. Cyanophyta were found, including one species from one genera, accounting for 1.7% of the total species, and chlorophyta were also found, including two species from two genera, accounting for 4.3% of the total species. Diatoms were the dominant species, including *Pseudonitzschia* (51.73%), *Skeletonema* (18.08%), and *Chaetoceros* (5.09%). *Dinophysis caudata* and *Dinophysis rotundata* (Table S2 in the Supplementary Material) were also found in the collected phytoplankton samples. Dinoflagellates, such as *Dinophysis* and *Prorocentrum*, are the mainly toxigenic algae producing OA, PTX2, and DTX1, which means *D. caudata* and *D. rotundata* are the possible main sources of OA, PTX2, and DTX1 in the seawater of the CJE. At present, the toxigenic algae that can produce OA, PTX2, and DTX1 in the coastal area of China mainly include *Dinophysis acuminata*, *Dinophysis fortii*, *D. caudata*, and *D. rotundata* [46,47]. *D. acuminata*, *D. caudata*, and *D. fortii* are found in the harmful algae bloom eruption area of the ECS in spring, and large amounts of PTX2, OA, and DTX1 were detected from these phytoplankton samples [41]. *D. acuminata*, *D. Caudata*, and *D. fortii* have also been detected in the large-scale dinoflagellate red tide area of the CJE and its adjacent ECS in spring [42]. These observations prove that the toxigenic *Dinophysis* exists extensively for a long time in the CJE and its adjacent ECS.

GYM, the cyclic imine compound, can stably exist in seawater for a long time [45]. As a kind of late-discovered LMAT, GYM has been detected in New Zealand [48] and Canada [49]. *Alexandrium ostenfeldii* and *Karenia selliformis* have been proven to produce GYM [50,51]. So far, no reports on the discovery of *K. selliformis* in the CJE and the ECS have been published, and the resting cysts of *A. ostenfeldii* have only been found in the sediment of the Bohai Sea [52]. In this survey, GYM was detected from seawater and phytoplankton samples of the CJE, which indicates that GYM-producing algae exist in the research area. However, there is no detection of *A. ostenfeldii* in the research area. Additionally, a phytoplankton species that is similar to *K. selliformis* in morphology, which is suspected to be a GYM-producing algae, was found among the collected phytoplankton samples and will be further studied.

### 2.6. Factors Affecting the Concentration and Distribution of LMATs in the CJE

To clarify the main factors affecting the distribution of LMATs in seawater from the CJE, the correlation between the concentration of LMATs and various physiochemical parameters of the surface seawater collected in August 2017 was analyzed. The results are shown in Table 1.

Here, Salinity (S) has a significant positive correlation with the concentrations of OA and PTX2 (Table 1). This finding indicates that the distribution of LMATs is consistent with that of S in the surface seawater of this area, and is supported by Figure 5 and Figure S4a (Supplementary Materials). Expansion of the CJDW toward the offshore area affects the growth of narrow salt phytoplankton in seawater, causing a low concentration of LMATs in the area covered by CJDW (Figure 5). Continuous expansion of the CJDW forms a distinct plume front interface (a salinity of 25 is usually defined as an inshore plume front interface parameter) that is in contact with the offshore seawater. In the plume front area (salinity of 20–27), the primary productivity is usually high (Figure S4a,f). However, the area with high concentration of LMATs occurs outside the boundary of the CJDW (salinity of 31), which indicates that the primary productivity is not correlated with LMAT concentration. The main reason may be that the abundance of toxigenic algae is not necessarily high in the waters of high primary productivity. Since the large number of phytoplankton may influence the growth and toxin production of toxigenic algae, the beneficial harm-avoidance behavior makes it move towards more favorable areas away from the high primary productivity area. Dissolved oxygen (DO) in seawater was positively correlated with the concentrations of OA, PTX2, and GYM (Figure 5 and Figure S4e). This result proves that increased DO in the surface seawater of the CJE is conducive to the growth of toxigenic algae and production of LMATs, consistent with a previous report [53]. The temperature of the seawater was negatively correlated with the concentration of OA and PTX2. As shown in Figure S4b (Supplementary Materials), the temperature of seawater in the CJE was generally high, at approximately 29 °C. This high temperature may be an important factor influencing the low LMAT concentration in the surface seawater of the CJE. Basti [54,55] found that the amount of LMATs produced by *D. caudata* and *D. acuminata* in room culture is significantly affected by temperature, because excessively high or low temperatures could inhibit the production of algal toxins. The concentration of some nutrients (e.g., dissolved inorganic nitrogen, silicate, and phosphate(P)) in seawater is also negatively correlated with the concentration of OA and PTX2 (Table 1). According to the rule of nutrient limitation for phytoplankton proposed by Justic [56], phosphorus limitation is the main nutrient status of phytoplankton in the CJE (Si: P > 2; DIN: P > 22) (Figure S5). During the sampling period, harmful algae bloom occurred near station B3, and the concentration of nitrate (Figure S4i–l) around the station significantly decreased due to the consumption of phytoplankton. This low-nitrogen and low-phosphorus (Figure S4h) environment could limit the growth of dominant diatoms in the late red tide outbreak period. Such a condition, however, is beneficial to the growth of dinoflagellates, such as toxigenic algae *Dinophysis*. Because of their strong ability to use organic nutrition, dinoflagellates can adapt to low inorganic nutrition environments [40,57,58]. Since the overall pH value in the research area does not fluctuate too much (Figure S4c), there is no significant correlation between pH and LMATs (including OA, PTX2, GYM and DTX1). Chla (Figure S4f) is an important parameter for evaluation of the biomass of phytoplankton. However, in the area with high phytoplankton biomass, the LMAT concentration is low. The reason may be that the growth of toxigenic algae is restricted in areas with large phytoplankton biomass, resulting in a small number of toxigenic algae and low toxin content. TSS content can indicate the degree of turbidity of seawater. The content of TSS decreased with the increase of offshore distance (Figure S4d). Although photosynthesis in phytoplankton is affected by the turbidity of seawater, the correlation data showed that the content of TSS was not the main factor affecting LMAT content. Therefore, there is no significant correlation between physiochemical parameters of pH, TSS, and Chla in seawater and LMATs (including OA, PTX2, GYM, and DTX1). The concentration of LMATs (OA and PTX2) in seawater is mainly affected by S, DO, temperature (T), dissolved inorganic nitrogen, silicate, and phosphate. Since few stations detected GYM and DTX1 and the concentrations observed were low, the correlation of GYM and DTX1 concentration with environmental parameters is not representative.

## 3. Conclusions

In this study, the composition, concentration, and distribution characteristics of LMATs in a typical large river estuary and its adjacent area were illuminated for the first time. PTX2, GYM, OA, and DTX1 were the main LMATs in the surface seawater and phytoplankton in this area. The concentration of LMATs in the research area reached to 105.54 ng/L and 3565.50 ng/g in seawater and phytoplankton, respectively. The concentration of LMATs can provide a basic data reference for future investigation of LMATs in coastal areas. The concentration of LMATs in the surface seawater initially increased with the offshore distance and then decreased, eventually forming a unique low-concentration zone covered by fresh water in the large river estuary, influenced by the CJDW. The unique distribution characteristics of LMATs in the large river estuary provide a scientific basis for the forecast and early warning of toxic harmful algal bloom. LMATs in the seawater of the research area were mainly derived from *D. caudata*, *D. acuminata*, and possibly, *K. selliformis*. Therefore, more research about LMATs can be focused on the *Dinophysis* spp. in the future. S and DO are positive factors that can affect LMAT distribution, while T and nutrient levels are negative factors affecting LMAT distribution. These results can provide scientific guidance for the protection of coastal aqueous environments and healthy development of marine fisheries.

## 4. Materials and Methods

### 4.1. Chemical Reagents

Certified reference materials of eight phycotoxins, namely OA, DTX1, YTX, AZA1, AZA2, GYM, SPX1, and PTX2, were purchased from the National Research Council, Institute for Marine Biosciences (Halifax, Nova Scotia, Canada). HPLC-grade acetonitrile and methanol were from Merck (Darmstadt, Germany). Liquid chromatography-mass spectrometry (LC-MS) grade ammonium hydroxide (≥ 25%) was obtained from Fluka (St. Louis, MO, USA). The water used in the experiments was pretreated by a Milli-Q water purification system (Millipore, Bedford, MA, USA).

### 4.2. Preparation of LMAT Standard Solutions

Standard stock solutions of OA, DTX1, YTX, AZA1, AZA2, GYM, SPX1, and PTX2 were prepared using methanol to concentrations of 200.00, 21.29, 245.83, 64.84, 61.21, 125.00, 352.87, and 220.35 μg/L, respectively. Working standard solutions were prepared by mixing these standards and diluting them with methanol. All standard solutions were stored at −20 °C before use.

### 4.3. Solid-Phase Extraction of Seawater Samples

Seawater samples were pretreated using the method developed by Chen [8]. Prior to loading, an Oasis hydrophile-lipophile balance (HLB) cartridge (200 mg, 6 mL, Waters, Milford, MA, USA) was successively activated with 3 mL of methanol and 3 mL of ultrapure water. A total of 300 mL of filtered seawater was loaded at a flow rate of 1 mL/min. After loading, the HLB cartridge was washed with 3 mL of 15% methanol solution (*v*/*v*), dried, and then eluted thrice with 3 mL of methanol solution (containing 1% ammonia solution, *v*/*v*). All of the eluent (9 mL) was obtained and dried in a vacuum rotator in a 40 °C water bath. After being dissolved in 1 mL of methanol, the obtained eluent was filtered using a 0.22 μm nylon film for purification and preserved at −20 °C.

### 4.4. Extraction of LMATs in Phytoplankton

Phytoplankton samples filtered onto the glass microfiber filters were freeze-dried and weighed. The filter was placed in a small beaker and 10 mL of methanol was added. After ultrasonic breaking for 10 min and ultrasonic extraction for 10 min, the extract was transferred to a rotary flask. Ultrasonic extraction was repeated twice, and the obtained extracts were combined for vacuum rotary evaporation at 40 °C until dry. Finally, dry residues were reconstituted with 1 mL of methanol, filtered using 0.22 μm nylon filter, and stored at −20 °C in the dark.

### 4.5. HPLC-MS/MS Analysis Methods

An Agilent 1200 series HPLC system (Wilmington, DE, USA) equipped with a vacuum degasser, quaternary pump, an autosampler, and an Agilent Extend-C18 column (3 mm × 150 mm, 3.5 μm) was employed for chromatographic separation. The column was kept at 20 ± 2 °C Ultra-pure water (A: containing 6.6 mmol/L ammonium hydroxide) and 90% aqueous acetonitrile solution (B: containing 6.6 mmol/L ammonium hydroxide) were used as mobile phases at a flow rate of 0.4 mL/min and injection volume of 10 μL. Gradient elution started with 20% B, increased to 30% B at 15 min, increased to 47.5% B at 20 min, increased to 100% B at 45 min, and held for 5 min.

An Agilent 6320 ion-trap mass spectrometer (Wilmington, DE, USA) equipped with an electrospray ionization (ESI) interface was employed for LMAT detection under the following conditions: electrospray ion source, atomizing gas pressure (N_2_), 40 psi; drying gas temperature, 350 °C; drying gas flow rate, 10 L/min; spray pressure, 241.3 kPa; and capillary voltage, 4.5 kV. MRM mode was used to detect the components of each LMAT using the time segment method. OA, PTX2, SPX1, AZA1, AZA2, DTX1, and GYM were detected by positive-mode electrospray ionization (ESI+), while YTX was detected in negative mode (ESI−). Details are shown in Table 2. The target compound was qualitatively validated by comparing the retention time and secondary mass spectrum of the characteristic ions of samples with those of the LMAT standards, and the external standard method was used for quantification.

### 4.6. Analytical Methods for Environmental Factors

Environmental factors of seawater samples collecgted in August 2017 were investigated, including T, S, pH, DO, TSS, Chla, and five dissolved nutrients: phosphate (PO_4_-P), silicate (SiO_3_-Si), nitrate (NO_3_-N), nitrite (NO_2_-N), ammonium (NH_4_-N), and dissolved inorganic nitrogen (DIN). Parameters of T and S were detected using a Seabird 911-Plus CTD (Seabird Electronics, Bellevue, WA, USA) in situ. Nitrate and nitrite concentrations were measured using the naphthalene ethylenediamine hydrochloride spectrophotometric method. Ammonium was measured using the phenol hypochlorite method. Phosphate was determined by phosphorus molybdenum blue spectrophotometry. Silicate was determined by silicon molybdenum blue spectrophotometry. These methods are all based on those used by Parsons [59]. Chla, which represents the phytoplankton biomass, was measured using a Turner Designs TD-700 fluorometer [59]. The pH was measured using a pHS-3C instrument, and DO was determined by electrochemical method based on the theory described by Parsons [59].

### 4.7. Statistical Analysis

Data of the sampling information were treated using Surfer 13 (Golden Software, Golden, CO, USA), and the concentrations of LMATs at each site were analyzed by the Kriging method to draw contour distribution maps. Correlation analysis was carried out using Statistical Package for the Social Sciences (SPSS) version 25 (IBM Corporation, Armonk, NY, USA). Matrices of correlations based on Pearson coefficients were sued to determine possible relationships between LMATs and environmental factors. Correlation coefficients (r) and probabilities (*p*) were determined to evaluate quality of fit. Differences were considered to be statistically significant when *p* < 0.05 and extremely significant when *p* < 0.01. To compare toxin concentrations among different sampling times and locations, the mean, interquartile range, maximum, and minimum values of toxin concentrations were determined; box plot figures were then prepared using SPSS 25.

### 4.8. Quality Assurance and Quality Control (QA/QC)

To ensure the reliability of the experimental results, the containers used were washed alternately with methanol and ultra-pure water 2–3 times before each experiment. Reagent and experimental condition blanks were used as controls. Reagent blanks, standard calibrations, and parallel samples were conducted once for every 10 samples to check whether the background values of the instrument were normal and to ensure the precision and accuracy of the experimental results. The detection limit (LOD) was three times the signal-to-noise ratio (S/N) of the instrument, and the quantitative limit was 10 times the S/N. The LODs of the eight LMATs were 0.028 ng/L (OA), 0.019 ng/L (PTX2), 0.019 ng/L (GYM), 0.071 ng/L (DTX1), 0.077 ng/L (YTX), 0.051 ng/L (AZA1), 0.008 ng/L (AZA2), and 0.023 ng/L (SPX1). The limit of quantitation (LOQs) of the eight LMATs were 0.093 ng/L (OA), 0.062 ng/L (PTX2), 0.064 ng/L (GYM), 0.236 ng/L (DTX1), 0.255 ng/L (YTX), 0.171 ng/L (AZA1), 0.027 ng/L (AZA2), and 0.078 ng/L (SPX1). The recoveries of the LMATs in seawater and phytoplankton ranged from 69.58%–92.07% and 89.68%–102.43%, respectively, and the relative standard deviation (RSD) of the repeatability of the method was in the range of 3.53%–9.88%.

## Figures and Tables

**Table 1:** Correlation coefficients were obtained from concentration values of LMATs (ng/L), values of temperature (T, °C), Salinity (S), dissolved oxygen (DO, mg/L), pH, total suspended substance (TSS, mg/L), Chla (μg/L), PO_4_ (μmol/L), SiO_3_ (μmol/L), NH_4_ (μmol/L), NO_2_ (μmol/L), NO_3_ (μmol/L), and dissolved inorganic nitrogen (DIN, μmol/L) in surface seawater samples from the Changjiang estuary in August 2017.

Variables	OA	GYM	PTX2	DTX1
T	−0.480 *	−0.258	−0.502 *	0.244
S	0.770 **	0.308	0.555 **	−0.186
DO	0.580 **	0.702 **	0.843 **	−0.104
pH	0.236	−0.176	−0.239	0.195
TSS	−0.265	−0.171	−0.278	0.105
Chla	−0.008	−0.021	0.107	0.037
PO_4_	−0.535 **	−0.306	−0.429 *	−0.118
SiO_3_	−0.805 **	−0.335	−0.566 **	0.253
NH_4_	−0.023	0.234	0.414 *	0.080
NO_2_	−0.089	−0.266	−0.125	0.146
NO_3_	−0.821 **	−0.348	−0.584 **	0.208
DIN	−0.823 **	−0.348	−0.583 **	0.210

Note: * Correlation is significant at the 0.05 level (two-tailed); ** correlation is significant at the 0.01 level (two-tailed).

**Table 2:** Mass spectrometric instrument conditions used to detect LMATs in the seawater and phytoplankton samples in multi-reaction monitoring (MRM) mode.

Compounds	Molecular Formula	Retention Time (min)	Segment Time (min)	Precursor Ion (*m*/*z*)	Qualitative/Quantitative Ion (*m*/*z*)	Collision Energy/V
OA^a^	C_44_H_68_O_13_	12.6	8.5–16.0	827.5 [M+Na]^+^	809.6/723.5	1.0
YTX^b^	C_55_H_82_O_21_S_2_	20.2	16.0–24.5	1141.7 [M-H]^−^	1123.7/1061.8	1.5
DTX1^c^	C_45_H_70_O_13_	19.2	16.0–24.5	841.5 [M+H]^+^	823.5/737.5	1.5
AZA1^d^	C_47_H_71_NO_12_	24.9	24.5–30.2	842.5 [M+H]^+^	824.4/806.4	0.9
AZA2^e^	C_48_H_73_NO_12_	25.6	24.5–30.2	856.5 [M+H]^+^	838.5/820.7	1.1
GYM^f^	C_32_H_45_NO_4_	32.0	30.2–34.8	508.4 [M+H]^+^	490.4/392.4	1.0
SPX1^g^	C_42_H_61_NO_7_	35.3	34.8–48.0	692.4 [M+H]^+^	674.4/656.4	1.1
PTX2^h^	C_47_H_70_O_14_	36.0	34.8–48.0	876.7 [M+NH_4_]^+^	805.7/823.6	1.0

Note: OA^a^ = okadaic acid; YTX^b^ = yessotoxin; DTX1^c^ = dinophysistoxin-1; AZA1^d^ = azaspiracid-1; AZA2^e^ = azaspiracid-2; GYM^f^ = gymnodimine; SPX1^g^ = 13-desmethyl spirolide C; PTX2^h^ = pectenotoxin-2.

## Supplementary Materials

The following are available online at https://www.mdpi.com/2072-6651/11/10/596/s1. Figure S1: Extracted ion chromatograms (EICs) and MS^2^ spectra of eight lipophilic marine algal toxins using high performance liquid chromatography-tandem mass spectrometry. (a1): EIC of AZA1; (a2): MS^2^ spectrum of AZA1; (b1): EIC of AZA2; (b2): MS^2^ spectrum of AZA2; (c1): EIC of YTX; (c2): MS^2^ spectrum of YTX; (d1): EIC of DTX1; (d2): MS^2^ spectrum of DTX1; (e1): EIC of GYM; (e2): MS^2^ spectrum of GYM; (e3): EIC of the daughter ion of GYM; (e4): MS^3^ spectrum of GYM; (f1): EIC of SPX1; (f2): MS^2^ spectrum of SPX1; (g1): EIC of PTX2; (g2): MS^2^ spectrum of PTX2; (h1): EIC of OA; (h2): MS^2^ spectrum of OA. OA = okadaic acid; YTX = yessotoxin; DTX1 = dinophysistoxin-1; AZA1 = azaspiracid-1; AZA2 = azaspiracid-2; GYM = gymnodimine; SPX1 =13-desmethyl spirolide C; PTX2 = pectenotoxin-2. Figure S2. Comparison of LMATs mass fraction in the surface water of each station during three cruises collected in August 2017, May 2018 and July 2018. (The red line represents the median, the box height represents the interquartile range IQR (25%–75%), the circles and small stars are outliers, and the black vertical line represents the range of 1.5 IQR within the maximum and minimum interquartile values)., Figure S3. Comparison of the average mass fraction of different LMATs in phytoplankton and seawater samples collected from station B3 in August 2017. Figure S4. The distribution characteristics of physio-chemical parameters: (a) Salinity (S) distribution characteristics; (b) Temperature (T) distribution characteristics; (c) pH distribution characteristics; (d) Total suspended substances (TSS) distribution characteristics; (e) Dissolve oxygen (DO) distribution characteristics; (f) chlorophyll a (Chla) distribution characteristics; (g), SiO_3_ distribution characteristics; (h) PO_4_ distribution characteristics; (i) DIN distribution characteristics; (j) NO_2_ distribution characteristics; (k) NO_3_ distribution characteristics; (l) NH_4_ distribution characteristics. Figure S5. Ratio of DIN, P, and Si in seawater samples of 15 stations collected during August 2017 from Changjiang estuary. Table S1. The composition and concentration of lipophilic marine algal toxins in seawater samples in CJK and its adjacent ECS area (ng/L). Table S2. Species and abundance of phytoplankton collected from the Changjiang Estuary of stations A9, A14, A15, B3, B4, B6, C4 in August 2017.
