# Supplementary material for: Distribution Characteristics and Environmental Control Factors of Lipophilic Marine Algal Toxins in Changjiang Estuary and the Adjacent East China Sea
Source: Toxins (Basel). 2019 Oct 12;11(10):596. doi: 10.3390/toxins11100596 (PMC6833110; doi:10.3390/toxins11100596)
Supplement: Supplementary file 1 [file toxins-11-00596-s001.pdf]

Supplementary Materials: Distribution characteristics and environmental control factors of lipophilic marine algal toxins in Changjiang estuary and its adjacent East China Sea

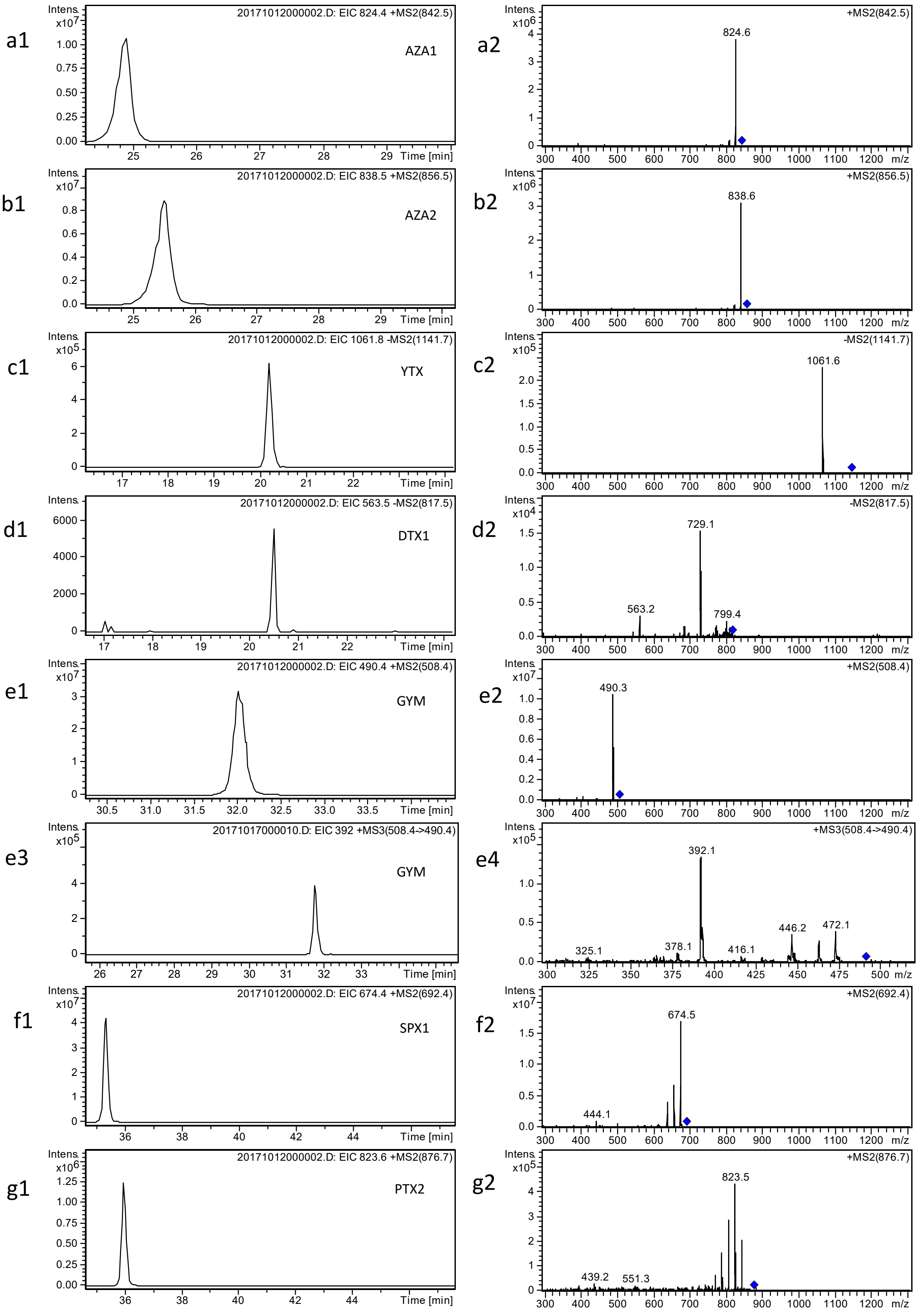

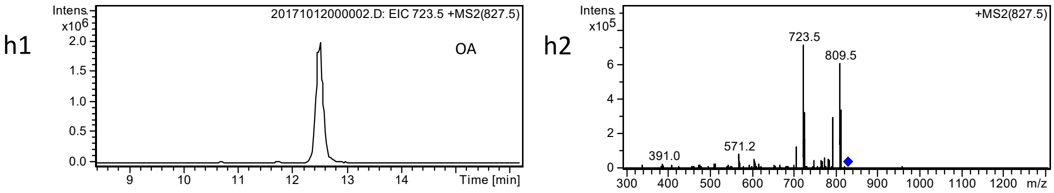

Figure S1. Extracted ion chromatograms (EICs) and MS^2 spectra of eight lipophilic marine algal toxins using high performance liquid chromatography-tandem mass spectrometry. (**a1**): EIC of AZA1; (**a2**): MS^2 spectrum of AZA1; (**b1**): EIC of AZA2; (**b2**): MS^2 spectrum of AZA2; (**c1**): EIC of YTX; (**c2**): MS^2 spectrum of YTX; (**d1**): EIC of DTX1; (**d2**): MS^2 spectrum of DTX1; (**e1**): EIC of GYM; (**e2**): MS^2 spectrum of GYM; (**e3**): EIC of the daughter ion of GYM; (**e4**): MS^3 spectrum of GYM; (**f1**): EIC of SPX1; (**f2**): MS^2 spectrum of SPX1; (**g1**): EIC of PTX2; (**g2**): MS^2 spectrum of PTX2; (**h1**): EIC of OA; (**h2**): MS^2 spectrum of OA. OA = okadaic acid; YTX = yessotoxin; DTX1 = dinophysistoxin-1; AZA1 = azaspiracid-1; AZA2 = azaspiracid-2; GYM = gymnodimine; SPX1 =13-desmethyl spirolide C; PTX2 = pectenotoxin-2.

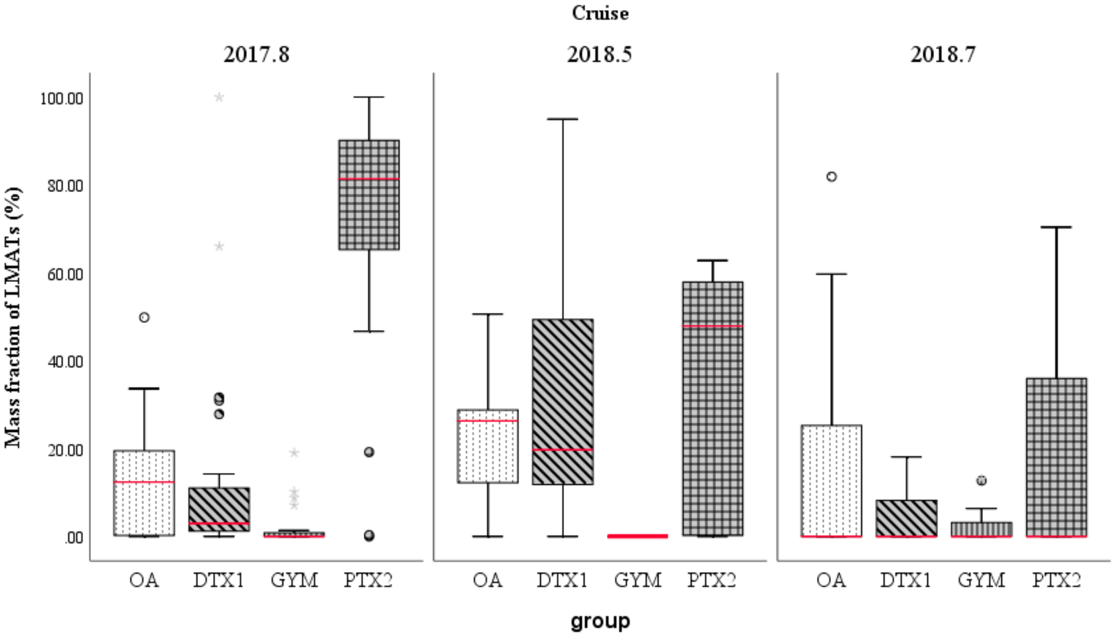

Figure S2. Comparison of LMATs mass fraction in the surface water of each station during three cruises collected in August 2017, May 2018 and July 2018. (The red line represents the median, the box height represents the interquartile range IQR (25%–75%), the circles and small stars are outliers, and the black vertical line represents the range of 1.5 IQR within the maximum and minimum interquartile values).

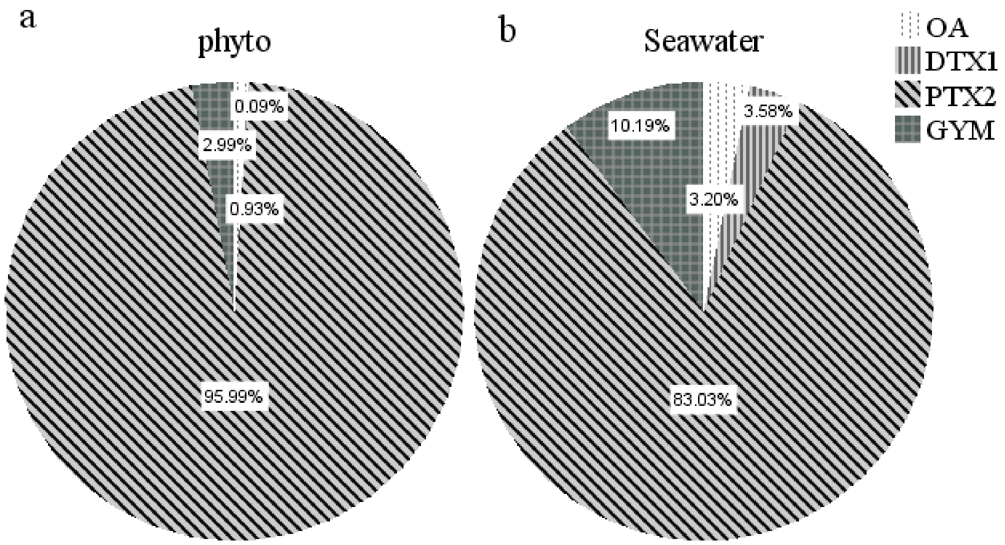

Figure S3. Comparison of the average mass fraction of different LMATs in phytoplankton (**a**) and seawater samples (**b**) collected from station B3 in August 2017.

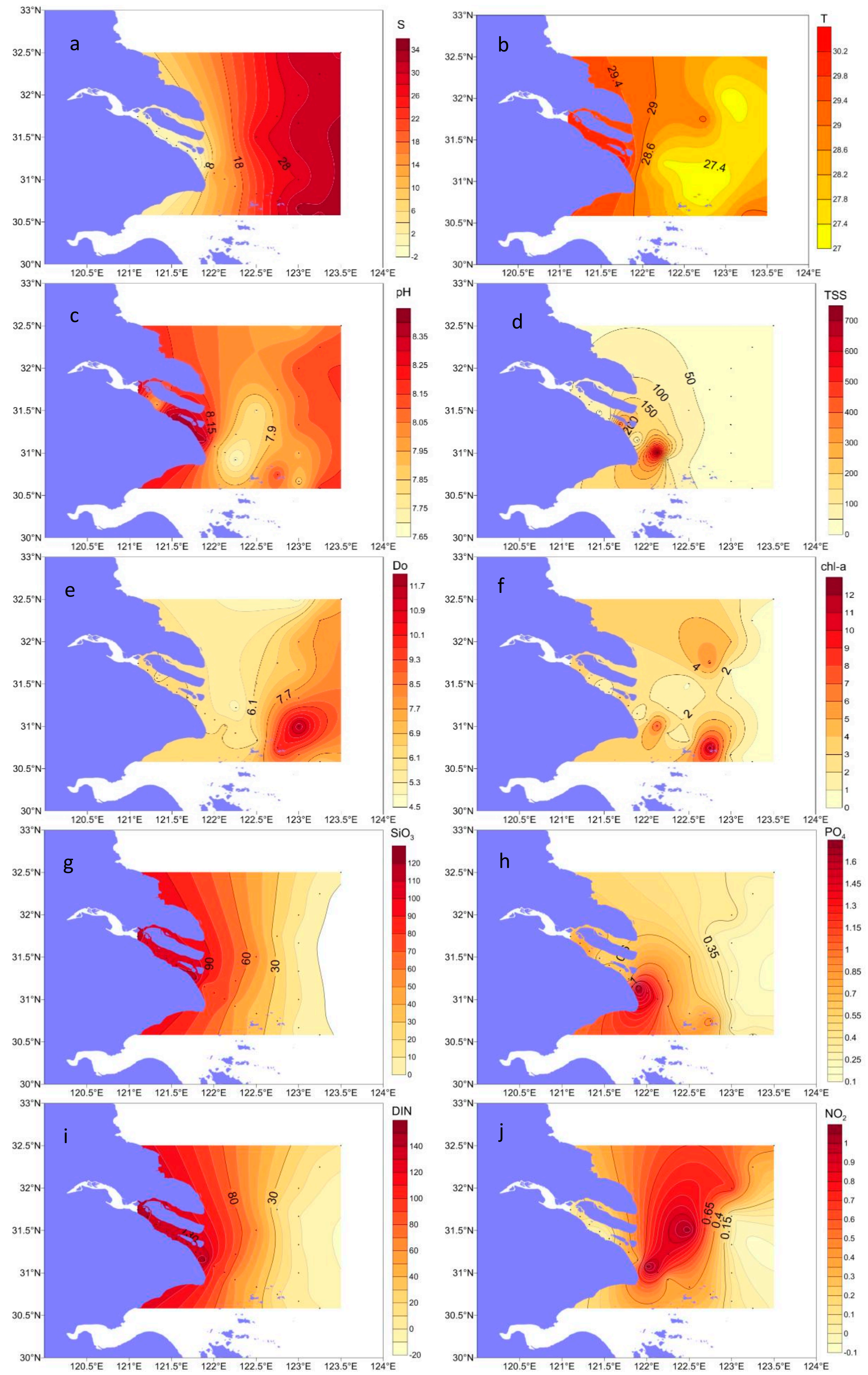
a
S
34
30
26
22
18
14
10
6
2
-2
8
18
28
b
T
30.2
29.8
29.4
29
28.6
28.2
27.8
27.4
27
c
pH
8.35
8.25
8.15
8.05
7.95
7.85
7.75
7.65
7.9
d
TSS
700
600
500
400
300
200
100
0
50
100
150
e
Do
11.7
10.9
10.1
9.3
8.5
7.7
6.9
6.1
5.3
4.5
f
chl-a
12
11
10
9
8
7
6
5
4
3
2
1
0
g
SiO3
120
110
100
90
80
70
60
50
40
30
20
10
0
h
PO4
1.6
1.45
1.3
1.15
1
0.85
0.7
0.55
0.4
0.25
0.1
0.35
i
DIN
140
120
100
80
60
40
20
0
-20
80
30
j
NO2
1
0.9
0.8
0.7
0.6
0.5
0.4
0.3
0.2
0.1
0
-0.1
0.65
0.4
0.15
33°N
32.5°N
32°N
31.5°N
31°N
30.5°N
30°N
120.5°E
121°E
121.5°E
122°E
122.5°E
123°E
123.5°E
124°E

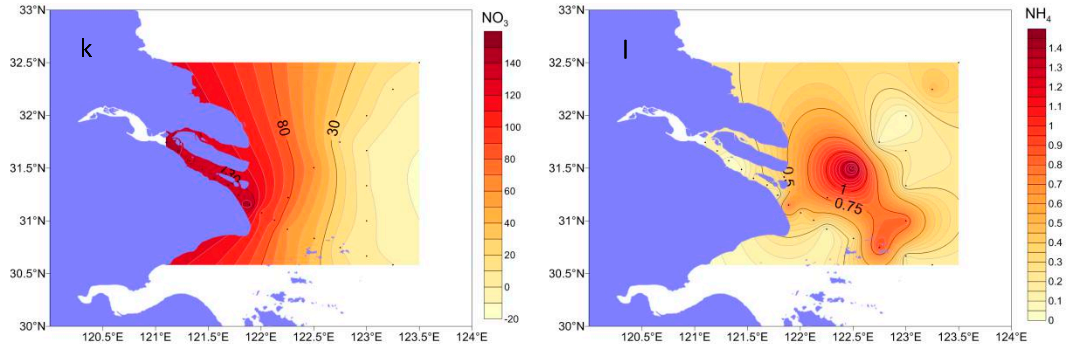

Figure S4. The distribution characteristics of physio-chemical parameters: (**a**) Salinity (S) distribution characteristics; (**b**) Temperature (T) distribution characteristics; (**c**) pH distribution characteristics; (**d**) Total suspended substances (TSS) distribution characteristics; (**e**) Dissolve oxygen (DO) distribution characteristics; (**f**) chlorophyll a (Chla) distribution characteristics; (**g**), SiO_3 distribution characteristics; (**h**) PO_4 distribution characteristics; (**i**) DIN distribution characteristics; (**j**) NO_2 distribution characteristics; (**k**) NO_3 distribution characteristics; (**l**) NH_4 distribution characteristics.

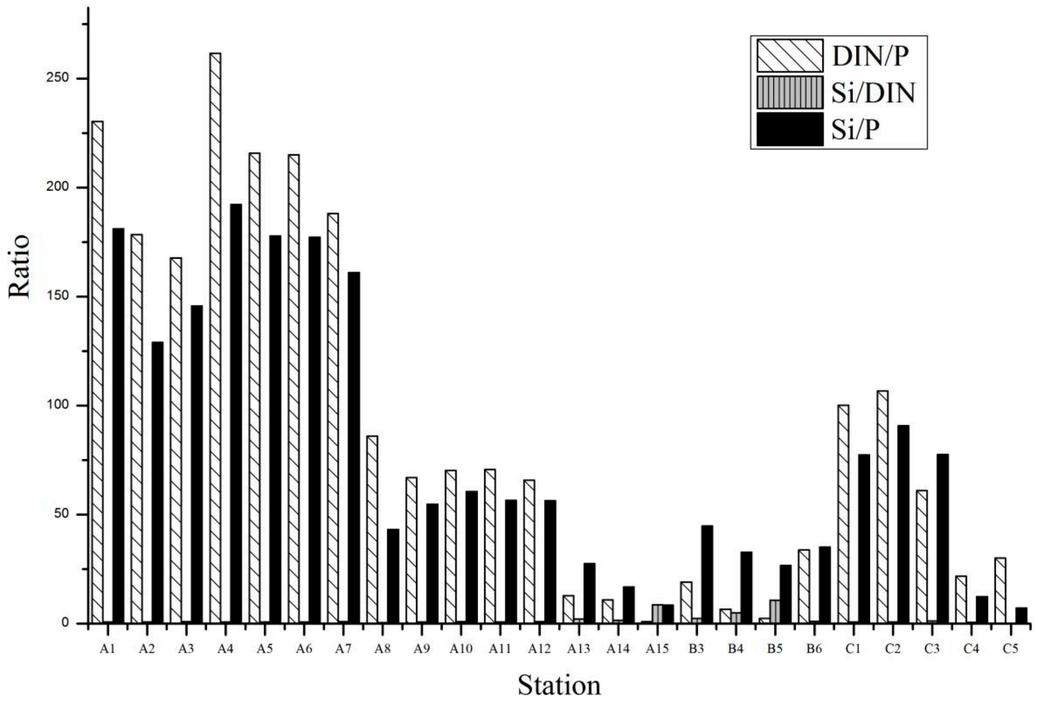

Figure S5. Ratio of DIN, P and Si in seawater samples of 15 stations collected during August 2017 from Changjiang estuary.

Table S1. The composition and concentration of lipophilic marine algal toxins in seawater samples in CJK and its adjacent ECS area（ng/L）.

Site	OA	GYM	PTX2	DTX1	Site	OA	GYM	PTX2	DTX1
A1	N.D.	N.D.	1.08	0.42	YT-3	2.78	N.D.	0.80	19.09
A2	N.D.	N.D.	1.02	N.D.	YT-2	16.13	N.D.	0.56	316.15
A3	N.D.	N.D.	0.56	N.D.	YT-1	14.42	N.D.	0.58	251.81
A4	N.D.	N.D.	N.D.	0.48	S01-1	1.07	N.D.	2.10	0.59
A5	N.D.	N.D.	2.34	N.D.	S01-4	1.37	N.D.	1.90	0.40
A6	N.D.	N.D.	2.20	0.36	S01-5	1.17	N.D.	2.64	0.75
A7	2.79	N.D.	0.03	5.48	S02-4	2.63	N.D.	2.81	3.68
A8	0.46	N.D.	0.99	0.67	S02-1	1.42	N.D.	2.18	1.35
A9	1.08	N.D.	0.41	0.67	S03-1	1.34	N.D.	2.93	1.27
A10	1.06	0.08	4.33	0.23	S04-5	bLOQ	N.D.	bLOQ	bLOQ
A11	1.29	N.D.	5.22	0.14	S04-1	2.19	N.D.	4.97	0.96
A12	1.70	N.D.	7.45	N.D.	S06-1	1.33	N.D.	3.51	0.75
A13	1.89	N.D.	34.45	0.68	S05-7	0.95	N.D.	bLOQ.	0.93
A14	2.84	1.60	17.59	0.45	S05-1	1.69	N.D.	3.18	0.56
A15	3.74	3.64	11.48	0.27	Average	3.46		2.01	42.74
B3	4.07	12.95	105.54	4.55	maximum	16.13		4.97	316.15
B4	2.65	0.29	25.71	0.81	K1	7.90	1.73	1.73	1.87
B5	3.06	1.36	10.32	0.67	K2	13.24	4.25	47.05	2.30
B6	2.02	0.11	14.51	N.D.	K3	10.35	6.97	34.41	3.33
C1	1.75	N.D.	3.89	0.48	K4	2.85	N.D.	5.46	0.96
C2	0.10	0.13	22.20	0.83	K5	1.54	N.D.	N.D.	0.34
C3	3.43	N.D.	17.64	0.63	K6	N.D.	N.D.	N.D.	N.D.
C4	2.67	1.70	62.88	0.69	K7	N.D.	N.D.	N.D.	N.D.
C5	3.19	N.D.	20.68	0.76	K8	N.D.	N.D.	N.D.	N.D.
Average	1.66	0.85	15.52	0.80	K9	N.D.	N.D.	N.D.	N.D.
maximum	4.07	12.95	105.54	5.48	K10	N.D.	N.D.	N.D.	N.D.
					K11	N.D.	N.D.	N.D.	N.D.
					K12	N.D.	N.D.	N.D.	N.D.
					Average	2.99	1.08	7.39	0.73
					maximum	13.24	7.00	47.05	3.33

N.D. represents not detected；bLOQ represents below LOQ.

Table S2. Species and abundance of phytoplankton collected from the Changjiang Estuary of stations A9, A14, A15, B3, B4, B6, C4 in August 2017.

Species	Abundance (cell/L)						
	**A9**	**A14**	**A15**	**B3**	**B4**	**B6**	**C4**
Bacillaria paxillifera	4	0	0	0	0	0	0
Bacteriastrum hyalinum	0	6992	0	1446	640	11412	460
Chaetoceros compressus	0	3128	16	904	0	0	0
Chaetoceros lorenzianus	4	0	8	246	0	0	0
Chaetoceros pseudocurvisetus	0	0	0	20	0	11040	84
Chaetoceros spp.	0	0	0	0	0	0	14352
Corethron hystrix	0	0	0	18	0	30	0
Coscinodiscus sp.	8	16	10	26	14	0	4
Coscinodiscus spinosus	0	4	0	10	0	368	6
Ditylum brightwellii	0	0	4	6	0	0	0
Guinardia delicatula	0	292	0	0	0	0	0
Guinardia flaccida	0	152	0	4	98	2208	48
Guinardia striata	2	242	8	556	640	13248	184
Hemiaulus sp.	0	0	2	0	0	108	0
Hemidiscus hardmannianus	0	4	0	2	0	14	0
Leptocylindrus danicus	0	918	0	436	368	4416	24
Odontella sinensis	0	0	0	2	0	0	0
Plerosigma affine	0	2	0	0	0	0	0
Pleurosigma pelagicum	2	4	0	0	0	0	0
Pseudo-nitzschia pungens	0	5336	460	27600	7360	141130	8832
Pseudosolenia calcar-avis	2	0	4	0	26	3312	3680
Rhizosolenia alate f. gracillma	0	12	0	0	10	3312	0
Rhizosolenia robusta	0	4	0	0	6	4	0
Rhizosolenia setigera	12	0	6	14	6	0	0
Rhizosolenia styliformis	0	0	2	0	0	84	16
Schrederella delicatula	0	420	0	512	42	2912	420
Skeletonema spp.	300	2460	9220	86	174	55200	0
Synedra sp.	4	0	0	0	0	0	0
Thalassionema nitzschioides	0	0	2	0	0	0	0
Thalassiosira eccentrica	2	0	0	0	0	0	0
Thalassiosira sp.	6	246	0	96	0	2900	84
Akashiwo sanguinea	0	0	0	276	26	2	0
Ceratium furca	0	442	16	150	482	1156	252
Ceratium fusus	0	4	14	12	270	120	4
Ceratium kofoidii	0	0	0	0	10	0	0
Ceratium lineatum	0	0	0	2	6	0	0
Ceratium trichoceros	0	0	6	0	12	0	0
Ceratium tripos	0	36	6	0	56	76	12
Dinophysis caudata	0	0	4	10	150	16	120
Dinophysis rotundata	0	0	0	0	2	18	24
Gonyaulax polygramma	0	0	0	84	324	56	12
Gonyaulax sp.	2	0	4	72	30	24	10
Heterocapsa spp.	0	6440	32	9200	9936	644	420
Karenia sp.	0	0	0	2392	0	108	0
Noctiluca scintillans	0	8	0	0	0	2	0
Pronoctiluca sp.	0	0	0	0	2	14	0

Prorocentrum micans	0	930	16	32	736	192	264
Protoperidinium bipes	0	0	6	14	12	30	0
Protoperidinium conicum	0	0	0	2	70	28	0
Protoperidinium elegans	0	6	2	8	74	24	0
Protoperidinium oceanicum	0	0	0	0	58	10	0
Protoperidinium pellucidum	0	0	8	38	920	0	0
Protoperidinium spp.	0	90	24	26	92	1800	920
Pyrophacus steinii	0	2	2	0	8	4	10
Scrippsiella trochoidea	0	736	62	64	134	112	0
Nostoc sp.	36	0	0	0	0	0	0
Lyngbya sp.	100	0	0	0	0	0	0
Pediastrum simplex	56	0	0	0	0	0	0
